# Retraction: Iptacopan Efficacy and Safety to Treat Paroxysmal Nocturnal Hemoglobinuria (PNH): A Systematic Review and Meta-Analysis

**DOI:** 10.7759/cureus.r163

**Published:** 2025-01-22

**Authors:** Ibrahim M Dighriri, Reham M Al-Qahtani, Amal O Almutairi, Rahaf N Alhashbari, Hanin A Kanbeja, Salman A AlOjaimi, Mona A Aljuaid, Abdullah A Albaradi, Sahar T Almanjumi, Samah A Alqurashi, Maryam S Majrashi, Hatoon M Alansari, Ghayah A Jabbari, Abdulaziz S Alharbi, Amnah A Alnami

**Affiliations:** 1 Department of Pharmacy, King Abdulaziz Specialist Hospital, Taif, SAU; 2 College of Pharmacy, King Khalid University, Abha, SAU; 3 Department of Pharmacy, East Jeddah General Hospital, Jeddah, SAU; 4 Department of Pharmacy, Ibrand Pharmacy, Taif, SAU; 5 Department of Pharmacy, Polyclinic of Dr. Zaher Qadeeb Alban, Makkah, SAU; 6 Department of Pharmacy, Dr. Suliman Alhabib Medical Group, Khobar, SAU; 7 College of Pharmacy, Taif University, Taif, SAU; 8 Department of Pharmacy, Zahrat-Alrawdah Pharmacy, Buraydah, SAU; 9 Department of Pharmacy, United Pharmacy, Taif, SAU; 10 Department of Pharmacy, United Pharmacy, Makkah, SAU; 11 Department of Pharmacy, Ibrand Pharmacy, Jazan, SAU; 12 College of Pharmacy, Jazan University, Jazan, SAU; 13 College of Pharmacy, Umm AlQura University, Makkah, SAU; 14 Department of Pharmacy, Sabya General Hospital, Gazan, SAU

This article has been retracted by the Editors-in-Chief due to extensive errors that cast doubt on the validity of the work. These errors include mislabeling congress abstracts (references 13, 14, 15 and 16) as "full-text" articles, the duplication of a study in the analysis, and incorrect graphs and tables that do not accurately correspond with the text. It has been determined that these errors exceed the limits of what could be reasonably addressed via correction. As a result, this article has been retracted.

